# *Boswellia serrata* Extract, 5-Loxin®, Prevents Joint Pain and Cartilage Degeneration in a Rat Model of Osteoarthritis through Inhibition of Inflammatory Responses and Restoration of Matrix Homeostasis

**DOI:** 10.1155/2022/3067526

**Published:** 2022-10-19

**Authors:** Mi-Rae Shin, Hwa-Young Kim, Hwang-Yong Choi, Kyoung Sik Park, Hak Joo Choi, Seong-Soo Roh

**Affiliations:** ^1^Department of Herbology, Daegu Haany University, Deagu 42158, Republic of Korea; ^2^Ju Yeong NS Co.,Ltd, Seoul 05854, Republic of Korea; ^3^Department of Biomedical Science, Cheongju University, Cheongju 42158, Republic of Korea

## Abstract

Osteoarthritis (OA) is a chronic, progressive joint disease associated with pain, functional impairment, and diminished quality of life in affected individuals. At a societal level, it also has a high economic burden. *Boswellia serrata* has been reported to have potent anti-inflammatory, antiarthritic, and analgesic effects. The aim of this study was to explore the therapeutic potential and possible underlying mechanism of 5-Loxin®, a standardized *Boswellia serrata* extract, in a rat model of OA. The OA model was established by the intra-articular injection of 50 *μ*L of monosodium iodoacetate (MIA) (60 mg/mL). 5-Loxin® was administered orally, and efficacy was evaluated through serum analysis, real-time polymerase chain reaction (PCR), histologic staining, and micro-computed tomography (micro-CT). Results indicated that administration of 5-Loxin® can relieve OA joint pain through inhibition of both inflammatory processes and cartilage degeneration. In the group of rats treated with 5-Loxin®, the suppression of inflammatory enzymes such as cyclooxygenase (COX)-2 and 5-lipoxygenase (LOX) resulted in a significant reduction in the prostaglandin (PG) E_2_ and leukotriene (LT) B_4_ levels. Moreover, 5-Loxin® ameliorated the deterioration of the main components of the articular extracellular matrix (ECM), such as glycosaminoglycans (GAGs) and aggrecan, through the downregulation of matrix metalloproteinases (MMPs). These findings suggest that 5-Loxin® may be a potential therapeutic agent for the treatment of OA.

## 1. Introduction

Osteoarthritis (OA), also known as degenerative arthritis, is the most common type of arthritis in adults. When OA occurs, joint cartilage wears out, causing pain, and bone deformation [1, 2]. OA is considered a non-inflammatory joint disease due to the absence of neutrophils in the synovial fluid. However, recent findings have challenged this view by demonstrating the involvement of inflammatory component-induced symptoms such as swelling, joint pain, and stiffness in OA. Therefore, inhibition of inflammatory pathways represents a new therapeutic strategy for OA [3].

OA is characterized by excessive and progressive degradation of the extracellular matrix (ECM), and pain in the affected joints [4]. Succinctly, the rate of ECM decomposition is faster than the production of the extracellular substrate surrounding the cartilage, disrupting the balance between the production and decomposition of cartilage substrate [5]. Moreover, the loss of aggrecan, a cartilage-specific proteoglycan core protein, and an increase in matrix metalloproteinases (MMPs), which degrade collagen, are closely associated with OA [6]. MMPs are endogenous proteolytic enzymes that play important roles in the degradation of various proteins and in tissue remodeling within the ECM. However, excess MMP activity can be destructive and can contribute to the pathogenesis of several conditions including arthritis, tumor invasion, and metastasis [7]. MMP expression is induced by inflammatory cytokines such as IL-1 and TNF-*α* which can be released by activated mononuclear cells, synoviocytes, or by the articular cartilage itself [8]. Suppression of proinflammatory cytokines and, therefore, MMPs, may be a valid approach to OA treatment.

Within inflammatory cells, arachidonic acid (AA) is metabolized to leukotriene (LT) B_4_ and prostaglandin (PG) E_2_ by lipoxygenases (LOXs) and cyclooxygenases (COXs), respectively. LTB_4_ is produced via the action of the 5-LOX enzyme. The 5-LOX activating protein (FLAP) facilitates the 5-LOX enzyme-mediated conversion of AA to pro-inflammatory LTB_4_ [9]. These eicosanoids are key pro-inflammatory mediators [10]. LTB_4_ is a powerful leukocyte activator that promotes adhesion and recruitment of leukocytes to the vascular endothelium. It also stimulates the production of proinflammatory cytokines such as IL-1*β* in macrophages and lymphocytes [11]. When the COX pathway is inhibited by indomethacin, AA metabolism primarily occurs via the 5‐LOX/LTB_4_ pathway. Indomethacin, a nonsteroidal anti-inflammatory drug, is used as a prescription medicine to reduce inflammation-induced pain, fever, stiffness, and swelling. However, long-term therapy is associated with a high incidence of wide-ranging adverse events such as bleeding, gastrointestinal ulcers, kidney disease, and heart attack [12]. Dual inhibition of both the COX and 5‐LOX pathways has shown additive therapeutic effects and improved safety compared with the use of COX inhibitors alone [13]. *Boswellia serrata*, a member of the Burseraceae family, is the best-known aromatic gum resin and was traditionally used in the Ayurvedic system of medicine. In some Asian countries, *Boswellia serrata* is widely used to control gum, mouth, throat complaints, and menstrual pain [14]. It has multiple therapeutic properties, including anti-inflammatory, bactericidal, antiatherogenic, anticancer, and analgesic effects [15]. In vitro studies have revealed that boswellic acids, including acetyl-11-keto-boswellic acid (AKBA), inhibit the biosynthesis of proinflammatory mediators such as LT and the proinflammatory transcription factor nuclear factor kappa B (NF-*κ*B) [16]. 5-Loxin® is a *Boswellia serrata* extract enriched with 30% AKBA. In the carrageenan-induced inflammatory model, 5-Loxin® treatment yielded significant improvements in paw inflammation in rats [17]. A previous in vitro study indicated that 5-Loxin® could potentially inhibit TNF-*α*-induced expression and activity of MMPs such as MMP-3, MMP-10, and MMP-12 in human microvascular endothelial cells [18]. Moreover, 5-Loxin® can inhibit 5-LOX and FLAP expression in LPS-stimulated THP-1 monocytes [19]. The efficacy and tolerability of 5-Loxin® were assessed in a previous double-blind, placebo-controlled clinical study [20]. Notably, 5-Loxin® treatment led to improved joint comfort and mobility 7 days post-treatment with both 100 mg and 250 mg doses. 5-Loxin® contains 30% AKBA, whereas Aflapin contains 20% AKBA-enriched nonvolatile oil [21]. These purified AKBA supplements decreased pain and enhanced physical functioning within a short period of time, with negligible side effects. Therefore, these supplements could help to reduce the economic burden associated with OA and improve the quality of life in affected patients [22]. In another study, which examined the effects of 5-Loxin® on OA of the knee joint, 5-Loxin® was well tolerated and exerted its therapeutic effect by reducing levels of synovial MMP-3 [23]. The aim of this study was to further investigate the therapeutic potential and underlying mechanisms of 5-Loxin® in a standardized MIA-induced OA rat model.

## 2. Materials and Methods

### 2.1. Materials

Monosodium iodoacetate and indomethacin (Cat Nos. I2512 and I7378) were purchased from Sigma-Aldrich Co. Ltd. (St. Louis, MO, USA). ELISA kits (MyBioSource, Inc., San Diego, CA, USA) were used to determine levels of rat aggrecan (MBS457470), rat glycosaminoglycan (GAGs; MBS7606342), rat cartilage oligomeric matrix protein (COMP; MBS009757), rat 5-lipoxygenase (5-LOX; MBS263096), rat 5-lipoxygenase activating protein (FLAP; MBS2515825), tumor necrosis factor-*α* (TNF-*α*; MBS175904), IL-1*β* (MBS175941), IL-6 (MBS175908), and rat COX-2 (cyclooxygenase-2; MBS020734). ELISA kits (R&D Systems, Inc., Minneapolis, MN, USA) were used to determine serum levels of prostaglandin E2 (PGE2; KGE004 B). ELISA kits (Elabscience Biotechnology, Inc.,Houston, TX, USA) were used to measure rat cross-linked C-telopeptide of type 2 collagen (CTX2; E-EL-R2554), rat MMP-2 (E-EL-R0618), rat MMP-9 (E-EL-R3021), and rat MMP-13 (E-EL-R0045). LTB_4_ was evaluated using an LTB_4_ parameter assay kit (KGE006 B; R&D Systems, Inc., Minneapolis, MN, USA).

### 2.2. Test Material

5-Loxin® (Fysolate Technologies, India) was provided by Ju Yeong NS Co. Ltd., Seoul, South Korea). 5-Loxin® is a *Boswellia serrata* extract enriched with 30% AKBA, using a selective enrichment process patented in India (#205269).

### 2.3. Development of OA with MIA Injections and 5-Loxin® Administration

A rat model of OA was induced by MIA, as previously described [24]. Seven-week-old male Sprague-Dawley rats (170–200 g; DBL Co., Eumseong, Korea) were housed three per cage with free access to food (ENVIGO, USA) and water, in a room with controlled temperature (22 ± 2°C), humidity (55 ± 5%), and lighting (12 h light/dark cycle, 200–300 Lux). After acclimatization (1 week), rats were assigned into five different groups (MIA-treated group, *n* = 10; normal, *n* = 8). Group one: distilled water was administered to normal rats; Group two: distilled water was administered to MIA control rats; Group three: indomethacin was administered to MIA rats (2 mg/kg); Group four: 5-Loxin® was administered to MIA rats (100 mg/kg); Group five: 5-Loxin® was administered to MIA rats (200 mg/kg). Dosage was determined with reference to previous studies [25, 26]. Experimental and control samples were administered orally (2 mL/day/animal) via a stomach tube for 2 weeks. OA was induced in the rats using the method described by Wang et al., with minor modifications [27]. Rats were anesthetized using an intraperitoneal injection of 0.75 mg/kg tiletamine and zolazepam (Zoletil; Virbac, France) and then injected with MIA (3 mg/50 *μ*L) through the patellar ligament, into the intra-articular space of the right knee using a 0.3-mL insulin syringe (31 *G* needle) [28]; normal rats were injected with an equivalent volume of saline. The hind paw weight-bearing ratio was used to determine whether OA had been induced. Two rats that did not develop OA were euthanized and the number of rats in each group was adjusted to 8. Groups were treated orally for 4 weeks, as described above. Rats were humanely euthanized at the end of the experimental period (isoflurane overdose whilst under anesthesia), and the right knee joints (including the femur and tibia) were separated for histological examination, western blot analysis, and micro-computed tomography. Excess soft tissue was carefully removed, and the articular surface was rinsed with phosphate-buffered saline. Blood was collected by cardiac puncture, centrifuged at 3,000 rpm for 15 min at room temperature, and serum was collected and stored at −80°C until analysis. All animal procedures were approved prior to the conduct of the study by the Animal Research Ethics Committee of Daejeon University (Permit Number: DJUARB 2019–003).

### 2.4. Measurements of Hind Paw Weight Distribution

OA induction leads to an imbalance in the weight-bearing ability of the hind paw. To assess inflammatory pain, weight-bearing was measured using an incapacitance meter (IITC Life Science, USA) [24]. The rats were placed in the supplied holders and allowed to adapt for approximately 5 min. The hind paws rested on two separate weight-averaging pads. As the rats shifted their weight from each pad, the instrument recorded their average weight. Three trials were conducted at 1 min intervals, and a ratio was calculated. A significant shift in weight from the arthritic site to the contralateral hind paw, that is, a weight-bearing deficit, was considered an index of pain. The ratio of hind paw weight-bearing was calculated using the following equation: (weight on right hind paw/weight on left hind paw) × 100.

### 2.5. Measurements of Serum Glycosaminoglycans and Aggrecan

ELISAs were performed according to the manufacturer's instructions. Serum standards (100 *μ*L) were applied to the wells of a 96-well plate and incubated at 37°C for 120 min. Plates were washed thrice with washing buffer, 100 *μ*L of biotin antibody was added, and the plates were incubated again for 60 min at 37°C. After washing, 100 *μ*L horseradish peroxidase (HRP) conjugate was added and incubated at 37°C for 60 min. After washing, 90 *μ*L of TMB substrate reagent was added and incubated at 37°C for 15 min, after which 50 *μ*L of the stop solution was added. Absorbance was determined at 405 nm using a microplate reader (Molecular Devices, SpectraMax, California, USA). The measured values were expressed as absolute values based on a standard curve.

### 2.6. Measurement of Serum COMP, CTX-II, MMP-2, MMP-9, MMP-13, 5-LOX, LTB_4_, FLAP, COX-2, PGE_2_, TNF-*α*, IL-1*β*, and IL-6

Serum standards (100 *μ*L) were applied to the wells of 96-well plates and incubated at 37°C for 90 min. Plates were washed thrice with washing buffer and 100 *μ*L of the detection antibody was added. Plates were incubated for 60 min at 37°C. After washing, 100 *μ*L of the HRP conjugate was added and incubated at 37°C for 30 min. After washing, 90 *μ*L of substrate reagent was added and incubated at 37°C for 15 min, followed by the addition of 50 *μ*L of stop solution. Absorbance was determined at 405 nm using a microplate reader. The measured values were expressed as absolute values based on a standard curve.

### 2.7. Isolation of mRNA and Real-Time Polymerase Chain Reaction (PCR) from Cartilage Tissue

Cold cell lysis buffer (1 mL; cat. 17221; iNtRON Biotechnology, Korea) was added to the cartilage tissue and homogenized. Chloroform (200 *μ*L; Sigma-Aldrich Co. Ltd., St. Louis, MO, USA) was added and the samples were mixed by vortexing. After centrifugation at 13,000 rpm for 10 min (Hanil, Supra R12, Gimpo, Korea), 400 *μ*L of the supernatant was collected. Subsequently, 400 *μ*L of binding buffer was added. The mixture was transferred to a column and centrifuged. Washing buffer A (700 *μ*L) was added to the column, centrifuged. The flow-through was discarded. 700 *μ*L buffer B was added, and the column was centrifuged again. Total RNA was eluted into fresh tubes in 50 *μ*L elution buffer. The RNA concentration and quality were evaluated using a nanodrop (Thermo Fisher, Massachusetts, USA). Reverse transcription was performed on 1 *µ*g RNA using AccuPower CycleScript (Bioneer Corp., Daejeon, Korea). The final reaction volume was adjusted to 20 *μ*L by adding DEPC-DW (Bioneer Corp., Daejeon, Korea) to the reaction mixture (reaction buffer, 100 mM DTT, 10 mM dNTPs, RNase inhibitor 20 units, stabilizer, and oligo dT20 primer). cDNA synthesis was performed for 60 min at 45°C using a single-temperature reaction, followed by heat inactivation for 5 min at 95°C (alpha cycler 1 PCRmax, Staffordshire, UK). Real-time PCR was performed on the cDNA. For mixing, 1 *μ*L of cDNA, 2 *μ*L of each primer (Table 1), 10 *μ*L of SYBR Green (Qiagen, Hilden, Germany), and 5 *μ*L of DEPC-DW were required. Following a 2 min denaturation cycle at 95°C, 40 cycles of 95°C for 5 s and 62.5°C for 30 s were performed. Gene expression levels were compared to controls.

### 2.8. Histological Examination and Microcomputed Tomography

The tissue was removed from the right femur and tibia prior to fixation in 10% formalin. The fixed tissues were submitted to KPNT (Chungbuk, Korea) for micro-computed tomography (micro-CT) and Safranin-O staining and analysis. Subsequently, the cartilage region was read by converting the micro-CT images into 3D images, and the stained tissue slides underwent histological examination under an optical microscope (Carl ZEISS, Axiolab, Jena, Germany). A quantitative analysis was performed using Image *J* (ver. 1.52, NIH, Bethesda, MD, USA).

### 2.9. Statistical Analysis

The data are expressed as the mean ± SD. Statistical comparisons were assessed by one-way ANOVA followed by Duncan's test (SPSS 21.0; SPSS Inc., Chicago, IL, USA). Statistical significance was set at *P* < 0.05.

## 3. Results

### 3.1. Effects of 5-Loxin® on the Ratio of Hind Paw Weight-Bearing

**Figure 1:**
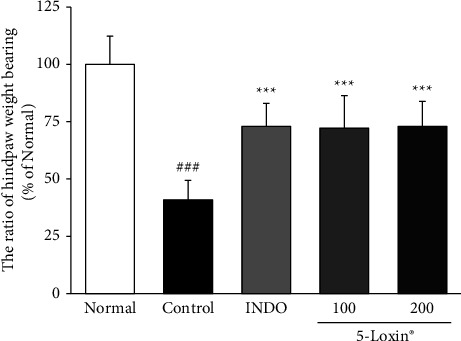
Effect of 5-Loxin on the ratio of hind paw weight bearing. The values represent the mean ± SD (*n* = 8). Normal: normal rats; control: MIA-induced osteoarthritis rats; INDO : MIA-induced osteoarthritis rats administered indomethacin (2 mg/kg body weight); 5-Loxin 100: MIA-induced osteoarthritis rats administered 5-Loxin (100 mg/kg body weight); 5-Loxin 200: MIA-induced osteoarthritis rats administered 5-Loxin (200 mg/kg body weight). Significance: ^###^*p* < 0.001*vs.* normal rat values and ^*∗∗∗*^*p* < 0.001*vs.* MIA control rat values.

Hind paw weight distribution (HWD) was measured using an incapacitance meter one day prior to the end of the experiment. Joint pain leads to decreased HWD. Both indomethacin- and 5-Loxin-treated animals had significantly better ratios of hind paw weight bearing than the MIA control animals, indicating that oral administration of test article 5-Loxin at doses of 100 and 200 mg/kg significantly reduced joint pain in a rat model of OA (Figure 1).

### 3.2. Effects of 5-Loxin on Matrix Homeostasis

**Figure 2:**
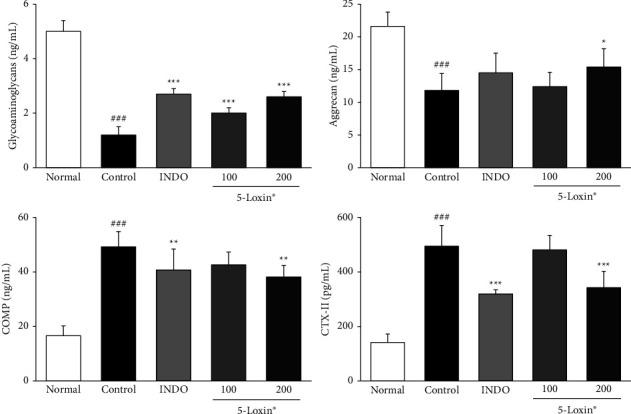
Effects of 5-Loxin on the serum level of glycosaminoglycans, aggrecan, COMP, and CTX-II. The values represent the mean ± SD (*n* = 8). Normal: normal rats; Control: MIA-induced osteoarthritis rats; INDO: MIA-induced osteoarthritis rats administered indomethacin (2 mg/kg body weight); 5-Loxin® 100: MIA-induced osteoarthritis rats administered 5-Loxin® (100 mg/kg body weight); 5-Loxin® 200: MIA-induced osteoarthritis rats administered 5-Loxin® (200 mg/kg body weight). Significance: ^###^*p* < 0.001*vs.* normal rat values and ^*∗*^*p* < 0.05, ^*∗∗*^*p* < 0.01, ^*∗∗∗*^*p* < 0.001*vs.* MIA control rat values.

**Figure 3:**
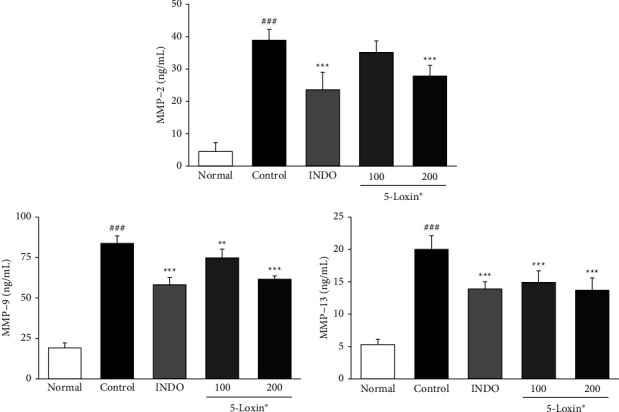
Effects of 5-Loxin on the serum levels of MMP-2, MMP-9, and MMP-13. The values represent the mean ± SD (*n* = 8). Normal: Normal rats; Control: MIA-induced osteoarthritis rats; INDO: MIA-induced osteoarthritis rats administered indomethacin (2 mg/kg body weight); 5-Loxin® 100: MIA-induced osteoarthritis rats administered 5-Loxin® (100 mg/kg body weight); 5-Loxin® 200: MIA-induced osteoarthritis rats administered 5-Loxin® (200 mg/kg body weight). Significance: ^###^*p* < 0.001*vs.* normal rat values and ^*∗∗*^*p* < 0.01, ^*∗∗∗*^*p* < 0.001*vs.* MIA control rat values.

**Figure 4:**
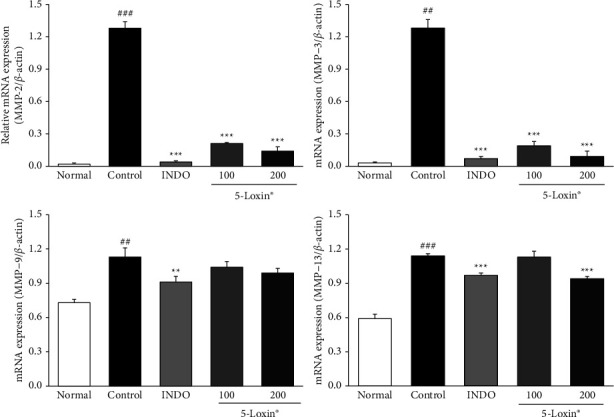
Effects of 5-Loxin on MMP-2, MMP-3, MMP-9, and MMP-13 gene expression levels in cartilage tissue. Values represent the mean ± SD (*n* = 4). Normal: normal rats; Control: MIA-induced osteoarthritis rats; INDO: MIA-induced osteoarthritis rats administered indomethacin (2 mg/kg body weight); 5-Loxin® 100: MIA-induced osteoarthritis rats administered 5-Loxin® (100 mg/kg body weight); 5-Loxin® 200: MIA-induced osteoarthritis rats administered 5-Loxin® (200 mg/kg body weight). Significance: ^##^*p* < 0.01, ^###^*p* < 0.001*vs.* normal rat values and ^*∗∗*^*p* < 0.01, ^*∗∗∗*^*p* < 0.001*vs.* MIA control rat values.

Figure 2 shows the effect of 5-Loxin on various molecules involved in matrix homeostasis, including glycosaminoglycans (GAGs), aggrecan, cartilage oligomeric matrix protein (COMP), and C-telopeptide of type II collagen (CTX-II). Both COMP and CTX-II biomarkers, which indicate cartilage breakdown, were significantly downregulated in 5-Loxin (200 mg/kg) treated animals compared to the MIA control group animals. A significant increase in GAGs was observed in the 5-Loxin (100 and 200 mg/kg) treated groups compared to the MIA control group, and aggrecan was also significantly increased in the 5-Loxin (200 mg/kg) treated group, relative to the MIA control group. Cartilage-degrading enzymes, including MMP-2, MMP-9, and MMP-13, were upregulated in MIA control rats relative to normal rats. These enzymes were significantly lower in the 5-Loxin (200 mg/kg) group than in the MIA control group (Figure 3). 5-Loxin (200 mg/kg) treatment significantly suppressed the MIA-mediated increases in MMP-2, MMP-3, and MMP-13 gene expression in the cartilage tissue (*p* < 0.001). While MMP-9 expression was not suppressed, there did appear to be a slight dose-dependent reduction of expression in 5-Loxin-treated animals (Figure 4).

### 3.3. Effects of 5-Loxin on Enzymes and Cytokines Involved in Inflammatory Responses

**Figure 5:**
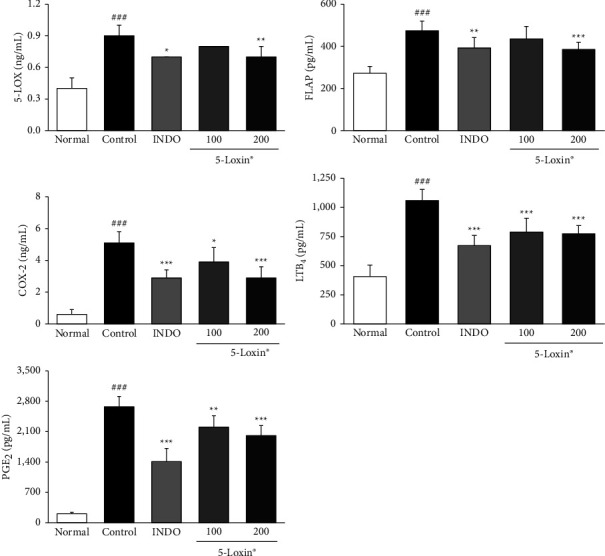
Effects of 5-Loxin on the serum levels of inflammatory markers (5-LOX, FLAP, COX-2, LTB_4_, and PGE_2_). The values represent the mean ± SD (*n* = 8). Normal: normal rats; Control: MIA-induced osteoarthritis rats; INDO: MIA-induced osteoarthritis rats administered indomethacin (2 mg/kg body weight); 5-Loxin® 100: MIA-induced osteoarthritis rats administered 5-Loxin® (100 mg/kg body weight); 5-Loxin® 200: MIA-induced osteoarthritis rats administered 5-Loxin® (200 mg/kg body weight). Significance: ^###^*p* < 0.001*vs.* normal rat values and ^*∗*^*p* < 0.05, ^*∗∗*^*p* < 0.01, ^*∗∗∗*^*p* < 0.001*vs.* MIA control rat values.

**Figure 6:**
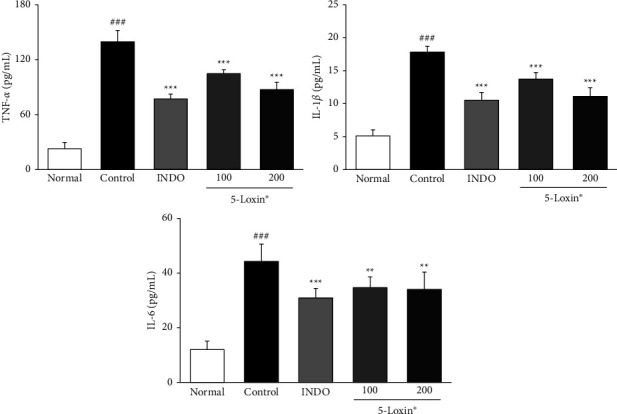
Effects of 5-Loxin on the serum levels of inflammatory cytokines (TNF-*α*, IL-1*β*, and IL-6). The values represent the mean ± SD (*n* = 8). Normal: normal rats; Control: MIA-induced osteoarthritis rats; INDO: MIA-induced osteoarthritis rats administered indomethacin (2 mg/kg body weight); 5-Loxin® 100: MIA-induced osteoarthritis rats administered 5-Loxin® (100 mg/kg body weight); 5-Loxin® 200: MIA-induced osteoarthritis rats administered 5-Loxin® (200 mg/kg body weight). Significance: ^###^*p* < 0.001*vs.* normal rat values and ^*∗∗*^*p* < 0.01, ^*∗∗∗*^*p* < 0.001*vs.* MIA control rat values.

**Figure 7:**
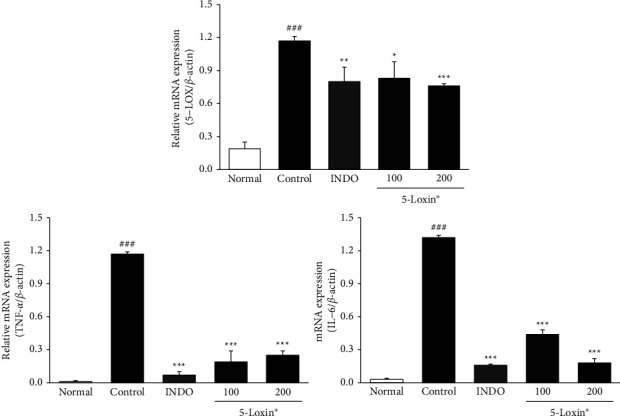
Effects of 5-Loxin on gene expression levels of TNF-*α*, 5-LOX, and IL-6 in cartilage tissue. Values represent the mean ± SD (*n* = 4). Normal: normal rats; Control: MIA-induced osteoarthritis rats; INDO: MIA-induced osteoarthritis rats administered indomethacin (2 mg/kg body weight); 5-Loxin® 100: MIA-induced osteoarthritis rats administered with 5-Loxin® (100 mg/kg body weight); 5-Loxin® 200: MIA-induced osteoarthritis rats administered 5-Loxin® (200 mg/kg body weight). Significance: ^###^*p* < 0.001*vs.* normal rat values and ^*∗*^*p* < 0.05, ^*∗∗*^*p* < 0.01, ^*∗∗∗*^*p* < 0.001*vs.* MIA control rat values.

Our data indicate that treatment with 5-Loxin (200 mg/kg) caused a reduction in the production of PGE_2_ and LTB_4_ via downregulation of both COX-2 and 5-LOX. Moreover, 5-Loxin (200 mg/kg) treatment downregulated levels of FLAP (Figure 5). Proinflammatory cytokines, such as TNF-*α*, IL-1*β*, and IL-6, are released during the inflammatory process, so we evaluated the anti-inflammatory effects of 5-Loxin by measuring their levels. TNF-*α*, IL-1*β*, and IL-6 levels were notably upregulated in the MIA control group. 5-Loxin treatment with 100 and 200 mg/kg significantly suppressed the release of proinflammatory cytokines (Figure 6). The severity of OA is modulated by the inflammatory processes that take place inside the cartilage tissue. These processes are mediated by proinflammatory cytokines such as TNF-*α*, IL-6 and inflammatory proteins such as 5-LOX. Elevated levels of TNF-*α*, 5-LOX, and IL-6 were significantly reduced by 5-Loxin treatment (Figure 7).

### 3.4. Histological Examination and Microcomputed Tomography

**Figure 8:**
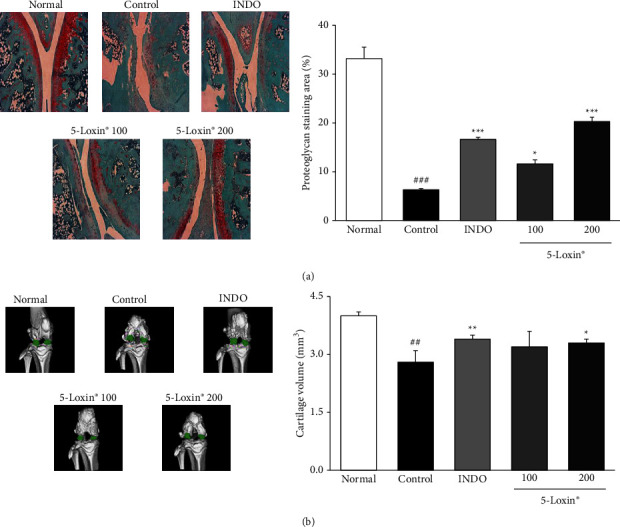
The representative images and quantitative evaluation of Safranin-O staining (magnification x200) (a) and micro-computed tomography (b) Normal: normal rats; Control: MIA-induced osteoarthritis rats; INDO : MIA-induced osteoarthritis rats administered indomethacin (2 mg/kg body weight); 5-Loxin® 100: MIA-induced osteoarthritis rats administered 5-Loxin® (100 mg/kg body weight); 5-Loxin® 200: MIA-induced osteoarthritis rats administered 5-Loxin® 200 mg/kg body weight. Significance: ^##^*p* < 0.01*vs.* normal rat values and ^*∗*^*p* < 0.05, ^*∗∗*^*p* < 0.01*vs.* MIA control rat values.

Micro-computed tomography has been extensively used to study microarchitectural changes in rat bones. In this study, micro-CT was used to measure the cartilage volume. MIA treatment has been shown to significantly reduce cartilage volume. This expected MIA-induced loss of cartilage volume was observed in the MIA-treated control rats, but supplementation with 5-Loxin (200 mg/kg) significantly reduced this loss of cartilage. This effect was also confirmed histologically (Figure 8). The loss of proteoglycan and structural changes, such as superficial leakage, observed in the MIA control were less severe in the 5-Loxin treated animals (Figure 8).

## 4. Discussion

Our findings revealed that 5-Loxin caused a significant reduction in inflammatory responses and cartilage degradation in aMIA-induced OA model in rats. One measure of OA in rats is reduced HWD due to joint pain and stiffness [29]. HWD assesses the distribution of the pressure exerted by each hind paw. Notably, when HWD was measured, the indomethacin and 5-Loxin (100 and 200 mg/kg) treated groups had a significantly higher HWD than in the MIA control group, indicating that there is reduced discomfort and stiffness in the 5-Loxin-treated animals.

The MIA effects seen histologically in this study concur with a recent study that observed similar structural changes, degeneration of articular cartilage, and loss of proteoglycans in MIA treated animals [30]. Herein, we demonstrated that 5-Loxin treatment significantly decreased cartilage damage and proteoglycan loss in MIA-induced OA, inhibiting these histological changes.

Biomarkers of degradation and inflammation in the blood, urine, and cartilage tissue can offer a measure of treatment efficacy or disease progression in clinical OA. Aggrecan and COMP are sensitive biochemical markers associated with the degradation of the articular cartilage that may be promising serum markers for monitoring OA [31]. CTX-II is another well-studied biomarker of cartilage breakdown [32]. GAGs are mucopolysaccharides that participate in various biological processes, including inflammation, bacterial and viral infections, and cancer. They play a major role in the regulation of leukocyte rolling and are involved in the downregulation of inflammatory processes [33]. These factors are precursors or products of metabolism that are released into the blood, synovial fluid, and urine, and their levels correlate with changes in OA. Previous studies have shown that the levels of GAGs and aggrecan were decreased in the MIA-treatedgroups, whereas COMP and CTX-II levels were increased after MIA treatment [34]. 5-Loxin (200 mg/kg) treatment counteracts these changes. Levels of GAGs and aggrecan were significantly increased relative to MIA control animals, whereas the levels of COMP and CTX-II were significantly lowered in the serum of 5-Loxin-supplemented animals. This reduction in the levels of cartilage breakdown markers demonstrates that 5-Loxin supplementation has beneficial clinical effects on OA.

Articular cartilage is composed of a specialized matrix of collagen, proteoglycans, and non-collagen components such as chondrocytes [35]. This balance can be disrupted due to ageing or joint disorders, and consequently, the rate of loss of collagen and proteoglycans from ECM may exceed the rate of production of newly synthesized molecules [36]. The loss of joint homeostasis because of an imbalance between the anabolic and catabolic processes is driven by inflammatory cytokine cascades during pathogenesis. Cartilage damage begins when proteoglycans are broken down by MMPs. Other inflammatory mediators, such as COX-2, 5-LOX, FLAP, PGE_2_, and LTB_4_, also drive OA progression. MMP-2 is a collagenase that is the main proteolytic enzyme among the MMPs. In addition to MMP-2, MMP-9, also known as gelatinase B, plays a role in ECM degradation. Both MMP-3 and stromelysin-1 can degrade a variety of ECM substrates, including collagen, laminin, fibronectin, osteopontin, and proteoglycans, while also demonstrating proteolytic activity on cell surface protein ectodomains [37]. High levels of MMP-13 and gelatinase can cause degradation of the basement membrane [38]. In this study, we demonstrated that 5-Loxin-mediated suppression of COX-2 and 5-LOX led to a significant decrease in PGE_2_ and LTB_4_. Moreover, the inhibition of inflammatory cytokines, including TNF-*α*, IL-1*β*, and IL-6, by 5-Loxin treatment resulted in a reduction in levels of cartilage-degrading enzymes, MMP-2 and 13, within both the serum and cartilage tissue. These results are consistent with the data reported in previous studies that showed treatment with 5-Loxin inhibited the expression of 5-LOX and FLAP in LPS-stimulated THP-1 monocytes and synovial MMP-3 in patients with OA.

One limitation of the current study was the absence of a quantitative evaluation of cartilage damage. Future studies will be required to clearly correlate the inhibition of inflammatory processes and clinical improvements observed during 5-Loxin treatment with a reduction in the cartilage damage index. This can be achieved through H&E staining, which can quantitatively evaluate cartilage damage.

## 5. Conclusions

This study demonstrated the therapeutic effects of 5-Loxin supplementation in relieving OA pain through the combined inhibition of pathogenic inflammatory processes and cartilage degeneration. Oral administration of 5-Loxin suppressed the production of inflammatory enzymes such as COX-2 and 5-LOX, resulting in a reduction of PGE_2_ and LTB_4_ production. Moreover, 5-Loxin reduced serum levels of MMPs, including MMP-2 and MMP-13, both of which are associated with cartilage degeneration during OA development, thereby indicating that in addition to alleviating pain, 5-Loxin could delay OA progression.

## Figures and Tables

**Table 1:** Primer sequences.

Gene	Forward (5′ ⟶ 3′)	Reverse (3′ ⟶ 5′)
MMP-2	GCACTGGTGTTGGGGGAGAT	GCTGCTGTATTCCCGACCAT
MMP-3	TTTGATGTACCCAGTCTACA	TCCAGAGAGTTAGATTTGGT
MMP-9	CCAGGAGTCTGGATAAGTTG	ACGCTCTGGGGATCCACCTT
MMP-13	TGACACCTCTGAATTTTACC	CCGCCAAGGTTTGGTCCAGG
5-LOX	GGCATGACTTTGCTGACTTT	TGCAGCGCTTGATGAGTACT
IL-6	TGAAGAACAACTTACAAGAT	ATTAGGAGAGCATTGGAAGT
TNF-*α*	CGAGTGACAAGCCCGTAGCC	TACAGCCCATCTGCTGGTAC
*β*-Actin	GACGGTCAGGTCATCACTAT	CGGATGTCAACGTCACACTT

## Data Availability

All data generated or analyzed during the current study are available from the corresponding author upon request.

## Supplementary Materials

Supplementary Materials Table S1. Measurements of body weight and food intake during the experiment.
