# Supplementary material for: Boswellia serrata Extract, 5-Loxin®, Prevents Joint Pain and Cartilage Degeneration in a Rat Model of Osteoarthritis through Inhibition of Inflammatory Responses and Restoration of Matrix Homeostasis
Source: Evid Based Complement Alternat Med. 2022 Oct 19;2022:3067526. doi: 10.1155/2022/3067526 (PMC9605825; doi:10.1155/2022/3067526)
Supplement: Supplementary Materials — Table S1. Measurements of body weight and food intake during the experiment. [file 3067526.f1.doc]

***Boswellia serrata* Extract, 5-Loxin, Prevents Joint Pain and Cartilage Degeneration in a Rat Model of Osteoarthritis through Inhibition of Inflammatory Responses and Restoration of Matrix Homeostasis**

**Supplementary Materials**

**Table S1**. Measurements of body weight and food intake during the experiment.

| Group | Body weight  (Drug treatment)  Initial (g) | Body weight (MIA induction plus Drug treatment) | | | Food intake  (g/day/rat) |
| --- | --- | --- | --- | --- | --- |
|  |  | Initial (g) | Final (g) | Weight gain (g) |  |
| Normal | 180.4 ± 6.8 | 251.9 ± 20.4 | 402.7 ± 23.6 | 222.4 ± 20.4 | 27.5 ± 6.4 |
| MIA-treated rats |  |  |  |  |  |
| Control | 178.9 ± 7.5 | 241.1 ± 11.2 | 399.6 ± 12.0 | 220.7 ± 15.0 | 25.9 ± 3.0 |
| INDO | 178.8 ± 5.2 | 234.6 ± 8.4 | 387.0 ± 22.1 | 208.2 ± 19.1 | 21.7 ± 0.9 |
| 5-Loxin 100 | 180.2 ± 4.9 | 242.4 ± 6.9 | 406.1 ± 14.2 | 225.9 ± 15.2 | 22.8 ± 2.9 |
| 5-Loxin 200 | 179.9 ± 7.1 | 239.8 ± 13.0 | 398.3 ± 23.1 | 218.4 ± 22.2 | 22.1 ± 3.0 |

Values are the mean± SD (n = 8).

Normal: normal rats; Control: MIA-induced osteoarthritis rats;

INDO: MIA-induced osteoarthritis rats were administrated with indomethacin 2 mg/kg body weight;

5-Loxin 100: MIA-induced osteoarthritis rats administrated with 5-Loxin 100 mg/kg body weight;

5-Loxin 200: MIA-induced osteoarthritis rats administrated with 5-Loxin 200 mg/kg body weight.
